# Technologies and therapeutics for ongoing prevention of respiratory infections

**DOI:** 10.1002/cti2.1442

**Published:** 2023-02-27

**Authors:** Shannon C David

**Affiliations:** ^1^ Environmental Chemistry Laboratory, School of Architecture, Civil and Environmental Engineering Ecole Polytechnique Fédérale de Lausanne (EPFL) Lausanne Switzerland

The coronavirus disease 2019 (COVID‐19) pandemic is the latest in a series of global high‐consequence respiratory virus outbreaks. In prior years, we faced the emergence of severe acute respiratory syndrome (SARS) during 2002–2003,^1^ swine‐like H1N1 influenza A virus in 2009^2^ and Middle East respiratory syndrome coronavirus (MERS‐CoV) during 2012.^3^ We continue to confront the seasonal recurrence of coronaviruses and influenza viruses, and our extensive agricultural practises and ongoing deforestation are considered major drivers of infectious zoonotic disease outbreaks. This, paired with the ever‐increasing globalisation of our world, means additional respiratory pandemics are a real possibility in our future.

For COVID‐19, early pandemic public health responses focussed on nonpharmaceutical interventions, and control measures were implemented globally to limit viral spread and slow down any potential overwhelming of healthcare systems. These interventions included border closures and travel restrictions, closing of day‐care centres, schools, restaurants and shops, the cancellation of mass events, mandatory wearing of face masks and numerous physical isolation measures. The near‐simultaneous global implementation of these nonpharmaceutical interventions helped to slow down the community transmission of SARS‐CoV‐2, mitigated the burden of disease on healthcare resources and allowed time to develop vaccines and treatments. An unexpected but positive phenomenon resulting from the mass implementation of these pandemic measures was the significant drop in non‐COVID‐19 respiratory viral infections and gastrointestinal viral infections globally.^4^, ^5^, ^6^, ^7^

An astounding decrease in influenza virus infections in both northern and southern hemispheres was by far one of the most noteworthy changes in non‐COVID‐19 disease incidence during the pandemic period.^8^, ^9^, ^10^ Comprehensive analysis of the GISRS FluNet database in a 2022 study^10^ showed that influenza cases sharply fell during the initial months of the pandemic to <100 cases per week. Compared with prepandemic numbers of ~50 000 cases per week during the 2018–2020 winter seasons, this constituted an unprecedented 99.8% reduction in incidence of influenza disease. Similarly, in the Southern Hemisphere, activity during the 2017–2019 winter seasons peaked between 1500 and 3500 positive cases per week, with a sharp decline following the start of the pandemic; influenza cases dropped to < 12 per week during May 2020 in this hemisphere, with cases remaining < 100 per week until November 2021.^11^ The reduction in community respiratory virus activity led to additional downstream effects, including associated decreases in secondary bacterial infections such as invasive pneumococcal diseases.^12^, ^13^, ^14^

With the progressive removal of pandemic measures, these decreases in disease incidence have not been sustained. For example, the partial relaxing of measures over the summer period between 2021 and 2022 caused an unseasonal spike in influenza cases in the Southern Hemisphere, and infection levels are now back to prepandemic levels.^11^ Spikes in infection with respiratory syncytial virus (RSV) were also reported in Australia upon relaxing of COVID‐19 restrictions.^7^ In northern areas, the most recent flu season arrived earlier than anticipated,^15^ and total case numbers were notably higher than in prepandemic years, with > 70 000 weekly cases reported at the season peak for 2022–2023.^11^ Prolonged suppression of seasonal influenza circulation during the 2020s is also expected to lead to greater ongoing susceptibility to respiratory infections in the birth cohort from this period, because of lack of natural exposures.^10^ Additionally, many young children missed out on important early childhood vaccines because of COVID‐related disruptions, and it is a priority to ensure the new generations are suitably protected from preventable diseases. To achieve this, focus should be given to enhancing the efficacy of vaccines currently available, and to the development of vaccines against pathogens not currently covered. In fact, recent studies report reductions in both activation and progression of non‐COVID19‐related clinical trials during the pandemic period,^16^, ^17^ because of difficulties in safely continuing under lockdown conditions, and a marked re‐orientation in clinical trial research towards COVID‐19.^18^ In this Special Feature of *Clinical & Translational Immunology*, we seek to highlight important non‐COVID‐19 vaccine papers from the past year that aimed at combating ongoing respiratory threats and improving protection in the coming years.

The Special Feature Review by Elkashif *et al*.^19^ summarises the knowledge to date on the molecular biology and immunology of the adenovirus‐based vaccine platform. This viral vector‐based vaccine delivery system is known to induce strong humoral and cell‐mediated immunity and is an attractive vaccine strategy in a pandemic scenario to rapidly produce large quantities of vaccine in a relatively short time frame. This review also describes the status of adenovirus‐based vaccines currently in preclinical or clinical studies, with focus on respiratory pathogens including influenza A virus, coronaviruses and RSV. Some of these Ad‐vectored vaccines in development are also intended for administration via the intranasal route for improved protection against mucosal pathogens. Here, immune induction would be mediated and/or aided by alveolar macrophages presenting antigens to T cells in lymph nodes draining from the respiratory mucosa, which may lead to immune memory at the site of pathogen encounter.

Similarly targeting intranasal delivery, the Special Feature Review by Williams *et al*.^20^ details a relatively new class of innate lymphoid cells (ILC2s) that play a crucial role in orchestrating protection against respiratory pathogens at the mucosa. Here, the authors describe the potential for lung ILC2s to induce mucosal immunity against influenza A viruses and discuss the possibility of targeting these cells as a type of innate adjuvant for enhanced delivery and immune processing of mucosal vaccines.

Finally, an Original Article from Ercoli *et al*.^21^ highlights vaccine considerations that must be made for individuals that are impaired in terms of immune‐competence. Immune‐compromised individuals are some of the most at‐risk of developing severe disease or complications because of infection with seasonal viruses and emerging pandemic pathogens, and thus must be protected as robustly as possible. Ercoli *et al*. specifically demonstrate how crucial the timing of vaccination is for individuals that are B‐cell‐depleted, to ensure sufficient vaccine‐mediated protection. B‐cell depletion is an effective therapy for autoimmune diseases and for B‐cell malignancies, but this therapy also leaves the patient highly susceptible to infections, particularly with respiratory pathogens. This effect is known and can often be intentionally countered with specific vaccinations around the time of depletion. Ercoli *et al*. investigated the effect of vaccinating either prior to or immediately after B‐cell depletion therapy and found that protection against the respiratory bacterium *Streptococcus pneumoniae* was most robust when vaccines were administered prior to depletion. Specifically, B‐cell depletion after Prevnar‐13 vaccination had little effect on both Prevnar‐induced serological responses and protection of animals against pneumococcal pneumonia. However, if a patient is unable to receive vaccinations because of safety concerns or clinical issues, Ercoli *et al*. also demonstrated that vaccination immediately after B‐cell depletion still offers partial protective efficacy, at least in the short term. This was not simply because of retained T‐cell immunity, as additional T‐cell depletion did not abrogate the ongoing partial protection against *S. pneumoniae*.

Overall, we have made remarkable progress over the last 2 years in creating new tools and improving our understanding of SARS‐CoV‐2 – knowledge that can now be applied to other respiratory infectious diseases. In addition to improved surveillance, communication plans, quarantine practises and public health protocols, the global deployment of effective vaccines is one of the most meaningful strategies we have to protect lives and limit global disease spread.
